# Epidemiology of paediatric trauma in Norway: a single-trauma centre observational study

**DOI:** 10.1186/s12245-019-0236-9

**Published:** 2019-07-31

**Authors:** Eirik Nesje, Nadine Nalini Valøy, Andreas Jorstad Krüger, Oddvar Uleberg

**Affiliations:** 1 Faculty of Medicine and Health Sciences, Norwegian University of Science and Technology, NO-7491 Trondheim, Norway; 2 Department of Emergency Medicine and Pre-Hospital Services, St. Olav’s University Hospital, NO-7006 Trondheim, Norway; 3 Department of Research and Development, Norwegian Air Ambulance Foundation, NO-0103 Oslo, Norway; 4 Department of Circulation and Medical Imaging, Norwegian University of Science and Technology, NO-7006 Trondheim, Norway

**Keywords:** Trauma systems, Trauma, Injury, Paediatric, Epidemiology

## Abstract

**Background:**

Trauma is a major cause of mortality and morbidity in children globally. The burden of injury shows substantial geographical differences, with a significant mortality reduction in children in Norway during the last four decades. The aim was to describe the current epidemiology, resource use and outcome for all potential severely injured paediatric patients admitted to a Norwegian trauma centre.

**Methods:**

This was a single-centre retrospective observational study. All patients aged 0–17 years received by a trauma team between 01 January 2004 and 31 December 2016 (13 years) at St. Olav’s University Hospital were included. Severe injury was defined as Injury Severity Score > 15.

**Results:**

A total of 873 patients were included, of which 536 (61%) were male. The median age was 13 years (IQR 7–16). Six per cent (*n* = 52) of the patients were transferred from other hospitals. Blunt trauma constituted 98%, with traffic (*n* = 532/61%) and falls (*n* = 233/27%) as the most common mechanisms. Eight patients (1%) died within 30 days of hospital admission. Fifteen per cent (*n* = 128) were severely injured. Among the patients transferred from another hospital, 46% (*n* = 24) had severe injuries. Helicopter Emergency Medical Services (HEMS) were more used in younger age groups and in patients more severely injured.

**Conclusions:**

In a developed healthcare system, the number of potentially severely injured children is small and with very few deaths following trauma. Transport and falls represent the most common causes of injury throughout all age groups, though with a tendency towards more transport-related injuries with increasing age. In-hospital trauma care is characterized by a low threshold for a multidisciplinary reception, low use of intensive care and need for emergency surgical procedures, though with increased need in the older children.

**Electronic supplementary material:**

The online version of this article (10.1186/s12245-019-0236-9) contains supplementary material, which is available to authorized users.

## Background

Trauma is a major cause of mortality and morbidity in children globally, with road injuries having the highest incidence rates [1]. The burden of injury experiences both substantial geographical and sociodemographic differences, with the highest incidence rates in low-income countries and lowest in the industrialized countries in Western Europe [1]. Also, within countries, the burden of injury is higher among children from low-income families [2].

Though the incidence of severe trauma among the paediatric population is declining in Europe, it still represents a major public health problem in the European Union [3]. As mortality rates have declined during the last two decades, morbidity after trauma is receiving more attention [4]. Many children, who survive a major trauma, are left with disabilities that affect their development, education and social life [2]. A report by the World Health Organization (WHO) showed that nearly 50% of children under the age of 12 presenting to an emergency department with injuries were left with some form of disability [2].

In Norway, the mortality rate has dropped dramatically since the early 1970s. While 37 per 100,000 boys aged 0–17 died from accidents in 1970, the same number had decreased to 2 per 100,000 in 2012 [5]. Injury-related deaths sustained at traffic accidents were most common [5]. A study published in 2012 by Kristiansen et al. also showed substantial geographical differences in the distribution of fatal trauma with the highest figures in rural areas [6].

Even though several publications describing trauma systems and traumatic injuries have been published in the last few years, there is still a lack of detailed clinical knowledge concerning the paediatric trauma population [6–9]. Previous Scandinavian epidemiologic studies on paediatric trauma also mainly focus on those with severe injuries, traumatic brain injuries and rates of mortality [7, 9]. No regional or national studies have been conducted on the broader segment of those potentially severely injured, which also incorporates those with potentially moderate injuries. The knowledge of paediatric trauma in Norway is mostly based on national white paper reports, which mostly contain national overall figures of patients treated and corresponding fatality rates [5, 10].

Epidemiologic baseline values are needed in order to evaluate the potential need for improved triage and preventive and rehabilitative measures. Therefore, the aim of this study is to give a detailed description of epidemiology, resource use and outcome for all potential severely injured paediatric patients being admitted to a regional trauma centre.

## Methods

The study is an observational retrospective analysis of prospectively collected data from a regional trauma centre in Central Norway. The study follows the “Strengthening the Reporting of Observational Studies in Epidemiology” (STROBE) recommendations for reporting of observational cohort studies [11].

### Study setting

St. Olav’s University Hospital (SOH) serves as a regional trauma centre for Central Norway, with a patient population of approximately 721,000 persons [12]. The regional Emergency Medical Coordination Centre (EMCC) staffed by specially trained nurses and paramedics activate the trauma team according to pre-defined triage criteria. These have previously been described [13]. The EMCC operator primarily activates the trauma team, but the trauma team leader is consulted in cases where the EMCC operators are uncertain, pre-defined criteria do not apply to the current situation or pre-hospital personnel do not provide adequate information. The trauma team consists of 12 mandatory and some facultative members. The team is one-tiered and group-paged upon activation and meets in the emergency department prior to admission of potentially severely injured patients. The EMCC dispatches and coordinates emergency medical service (EMS) and Helicopter Emergency Medical Services (HEMS) within the region. Regular ambulance crews and paramedics provide basic pre-hospital care, and an anesthesiologist/paramedic crew responds separately by helicopter or rapid response vehicle when needed. St. Olav’s University Hospital receives patients in need of special surgical treatment (neuro- and cardiothoracic surgery) or multidisciplinary intensive care admitted directly or transferred from local hospitals.

### Data collection

All children aged 0 to 17 years who were received by the trauma team at St. Olav’s University Hospital (SOH) in the time period from 01 January 2004 to 31 December 2016 (13 years), alive on admission and registered in the trauma registry were included. Patients admitted to other hospitals in the region and subsequently transferred to the regional trauma centre fulfilling the above inclusion criteria were also included. SOH functions both as a local hospital for the city of Trondheim and surrounding areas and is the regional trauma centre in Central Norway. We assumed that most potentially severely injured children would be transported directly or transferred from local hospitals following initial resuscitation. Patients pronounced dead before hospital arrival were excluded. Potentially severely injured patients are defined as patients received by a trauma team upon arrival. Injury Severity Score (ISS) and New Injury Severity Score (NISS) were used to assess injury severity [14, 15]. The ISS and NISS use the Abbreviated Injury Scale (AIS) methodology, where each injury is assigned a severity code and a body region. The ISS score is the sum of the square of the AIS score of the three most severe injuries in the six ISS body regions and ranges from 0 (minor) to 75 (worst outcome). NISS incorporates the three most severe injuries regardless of the body region [15]. With this modification, the predictive ability in penetrating trauma and isolated head injury is improved [16]. We defined major trauma/severe injury as patients having an Injury Severity Score (ISS) above 15, as ISS > 15 has been reported to predict a mortality rate of at least 10% [17, 18]. We defined a moderate injury as an ISS 9–15 and minor injury with an ISS 0–8.

A specially trained nurse prospectively collected all the data and documented the data in paper charts during trauma team examination at SOH. These data were then registered in the local trauma registry. During the study period, two databases were used to capture the entire study period, from 01 January 2004 to 31 December 2013 and from 01 January 2014 to 31 December 2016. Some definitions may be different between the two databases. In cases where the data could not be adequately defined, these events were described as “unspecified”. We extracted these anonymously clinical data retrospectively for study purposes. An overview of the collected variables is shown in Additional file 1: Table S1.

### Statistics

Descriptive characteristics of the study sample are presented as medians with interquartile ranges (IQR) and as absolute numbers, percentages and ranges. Data analysis was performed using SPSS statistical software (IBM Corporation, released in 2015; SPSS Statistics for Windows, Version 22, IBM Corporation, Armonk, NY, USA).

### Ethics

The regional ethics committee (REC) was informed about the study and deemed the study a clinical quality study not needing formal REC approval (reference number 2017/842/REC Central). The study received institutional approval (reference number ESA 17/5481) and approval by the Norwegian Centre for Research Data (reference number 55283/dated 12.10.2017).

## Results

A total of 873 patients were included, of which 536 (61%) were male. Children aged 0–17 years constituted 17% (873/5013) of all patients treated by the trauma team in the study period. The median age was 13 years (IQR 7–16). Six per cent (*n* = 52) of the patients were transferred to the trauma centre from a local emergency hospital, with the remaining 94% (*n* = 821) being transported directly from the scene (Table 1). Blunt trauma was the dominating type of injury in 851 patients (98%), and traumas related to traffic (*n* = 532/61%) and falls (*n* = 233/27%) were the most common injury mechanisms. Eight patients (1%) died within 30 days of hospital admission. In average, there were 67 patients that fulfilled the inclusion criteria each year, with an average of 10 severely injured (ISS > 15) children annually. The quality of the collected study variables showed few missing values, with the exception of physiological values, discharge status and computer tomography (CT) findings (Additional file 1: Table S1).

**Table 1:** Baseline patient characteristics

	All patients	Direct admissions	Transfers from local hospital	ISS > 15
Total, *n* (%)	873 (100)	821 (94)	52 (6)	128 (15)
Mechanism of injury, *n* (%)				
Transport	532 (61)	505 (62)	27 (52)	77 (60)
Violence	28 (3)	24 (3)	4 (8)	5 (4)
Fall	233 (27)	217 (26)	16 (31)	28 (22)
Others	79 (9)	75 (9)	4 (8)	18 (14)
Unknown	1 (< 1)	0	1 (2)	0
Injury Severity Score (ISS), *n* (%)				
Minor injury ISS 1–8	599 (68)	590 (72)	9 (17)	–
Moderate injury ISS 9–15	146 (17)	127 (15)	19 (37)	–
Severe injury ISS > 15	128 (15)	104 (13)	24 (46)	–
New Injury Severity Score (NISS), *n* (%)				
Minor injury NISS 1–8	573 (65)	565 (69)	8 (15)	–
Moderate injury NISS 9–15	129 (15)	117 (14)	12 (23)	–
Severe injury NISS > 15	171 (20)	139 (17)	32 (62)	–
Intubated prior to hospital arrival, *n* (%)				
No	776 (89)	752 (91)	24 (46)	76 (60)
Yes	54 (6)	48 (6)	6 (12)	35 (27)
Unknown/missing	43 (5)	21 (3)	22 (42)	17 (13)
Patients on ventilator, *n* (%)	86 (10)	72 (9)	14 (27)	51 (40)
Patients admitted to ICU, *n* (%)	203 (23)	180 (22)	23 (44)	70 (55)
Length of stay				
< 3 days	566 (65)	556 (68)	10 (19)	17 (13)
> 3 days	307 (35)	265 (32)	42 (81)	111 (87)

### Injury severity

**Fig. 1:**
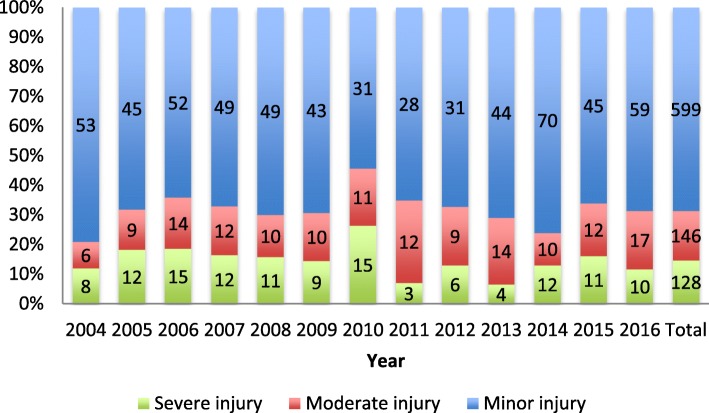
Injury severity and temporal distribution from 2004 to 20016. The figure illustrates the temporal development and injury severity throughout the study period. Figures inside the columns represent the exact number of cases dependent on injury severity and year. Severe injury is defined as ISS > 15, moderate injury as ISS 9–15 and minor injury as ISS 0–8

The Injury Severity Score (ISS) ranged from 0 to 54 (IQR 1–9), with a median of 4. Boys had a median ISS of 5; meanwhile, among girls, the median was 2. In all patients, 128 (15%) patients were severely injured, whereas 599 patients (69%) only had a minor injury (ISS < 9) (Tables 1 and 2). However, 171 (20%) patients had an NISS of more than 15. Among patients transferred from other hospitals, 46% (*n* = 24) had severe injuries (ISS > 15). The median ISS in patients that died (*n* = 8) was 26 (range 2–41). A patient with an ISS of 2 died within 30 days of the accident but after hospital discharge. We observed no clear trend development during the study period (Fig. 1).

**Table 2:** Patient characteristics of included patients, dependent on age groups

	All ages	0–4 years	5–9 years	10–14 years	15–17 years
Total, *n*	873	130	155	275	313
Injury Severity Score (ISS)					
Median (IQR)	4 (1–9)	2,5 (1–9)	4 (1–9)	5 (1–10)	4 (1–10)
Minor injury ISS 1–8, *n* (%)	599 (68)	94 (72)	110 (71)	185 (67)	210 (67)
Moderate injury ISS 9–15, *n* (%)	146 (17)	22 (17)	25 (16)	54 (20)	45 (14)
Severe injury ISS > 15, *n* (%)	128 (15)	14 (11)	20 (13)	36 (13)	58 (19)
New Injury Severity Score (NISS)					
Median (IQR)	4 (1–12)	3 (1–9)	4 (1–10)	5 (1–12)	5 (1–14)
Minor injury NISS 1–8, *n* (%)	573 (65)	92 (71)	103 (67)	175 (63)	203 (65)
Moderate injury NISS 9–15, *n* (%)	129 (15)	17 (13)	27 (17)	46 (17)	39 (12)
Severe injury NISS > 15, *n* (%)	171 (20)	21 (16)	25 (16)	54 (20)	71 (23)
Type of transportation to hospital					
EMS, *n* (%)	474 (54)	62 (48)	88 (57)	141 (51)	183 (59)
HEMS, *n* (%)	341 (39)	60 (46)	58 (37)	109 (40)	114 (36)
Others, *n* (%)	21 (3)	4 (3)	4 (3)	9 (3)	4 (1)
Unknown/missing, *n* (%)	37 (4)	4 (3)	5 (3)	16 (6)	12 (4)
Days in hospital total, *n* (median)	4075 (2)	520 (1)	622 (2)	1050 (2)	1883 (1)
Days in ICU total, *n* (median)	656 (0)	106 (0)	56 (0)	179 (0)	315 (0)
Days on ventilator total, *n* (median)	467 (0)	48 (0)	19 (0)	86 (0)	314 (0)
Inter hospital transfers, *n* (%)	52 (6)	7 (5)	5 (3)	25 (9)	15 (5)
Mechanism of injury, *n* (%)					
Transport	532 (61)	61 (47)	101 (65)	155 (56)	215 (69)
Violence	28 (3)	5 (4)	2 (1)	10 (4)	11 (4)
Fall	233 (27)	53 (41)	43 (28)	76 (28)	61 (19)
Others	79 (9)	11 (8)	9 (6)	33 (12)	26 (8)
Unknown/missing	1 (< 1)	0	0	1 (< 1)	0
First key emergency intervention, total *n*					
None	808	119	147	256	286
Damage control thoracotomy	3	0	1	1	1
Damage control laparotomy	16	3	2	6	5
Limb revascularization	2	0	0	1	1
Interventional radiology	2	0	0	0	2
Craniotomy	10	1	1	5	3
Intracranial pressure device insertion	17	3	3	4	7
Unspecified emergency intervention	6	0	0	2	4
Unknown	9	4	1	0	4
Intubated prior to hospital arrival, *n* (%)	54 (6)	10 (8)	11 (7)	13 (5)	20 (6)
Thoracic drainage, *n* (%)	22 (3)	2 (2)	3 (2)	5 (2)	12 (4)

### Age distribution

The median age in male patients was 12, and 13 years in female patients. The majority of the patients were among the older age groups, with 67% in the age groups 10 to 17 years (Table 2).

### Mechanism of injury

**Fig. 2:**
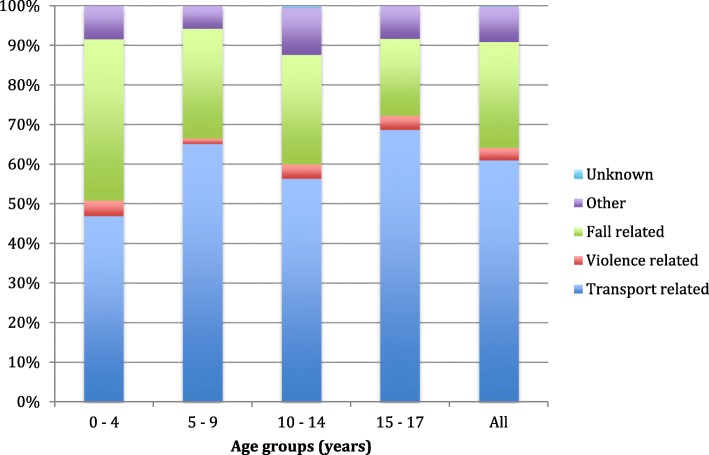
Mechanism of injury in different age groups. The figure illustrates the main mechanisms of injury within different age groups. Falls are more prevalent in the younger age groups, with a trend towards increased traffic-related injuries in the oldest age groups

**Fig. 3:**
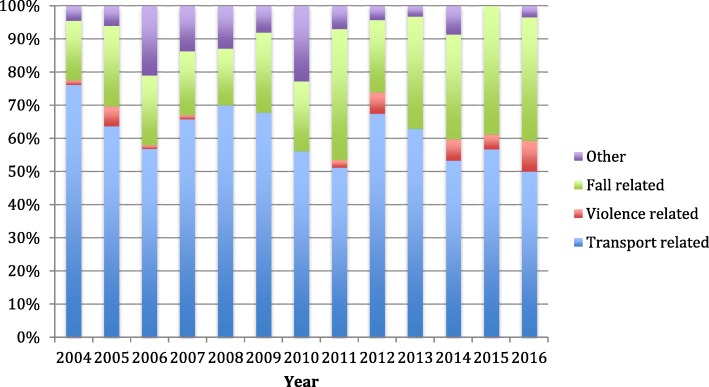
Development trends of the mechanism of injury. The figure illustrates the main mechanisms of injury and development throughout the study period. There is a trend towards fewer traffic-related injuries and an increased ratio of falls

Traffic-related accidents were the dominant mechanism of injury with 61% (*n* = 532) of all patients, followed by falls (27%, *n* = 233). In the age group 0–4 years, traffic-related injuries accounted for 47% (*n* = 61); meanwhile in the age group 15–17 years, 69% (*n* = 215) of injuries were traffic-related (Table 1; Fig. 2). Violence-related injuries had the highest median ISS of 8, while traffic-related injuries had the lowest of 3. For penetrating injuries (2% of patients), the median ISS was 10, while in blunt trauma (98% of patients), the median ISS was 4. In the minor injury group, 30% (*n* = 177) of the patients were injured in a car, followed by 27% (*n* = 163) who were injured by falls and 11% (*n* = 68) who were injured while on a motorbike. In the moderate injury group, the most common mechanisms of injury were falls (*n* = 42, 29%), car accidents (*n* = 24, 16%) and motorbike accidents (*n* = 17, 12%). The mechanisms of injury among non-survivors were car (*n* = 2), bicycle (*n* = 2), others (*n* = 2), falls (*n* = 1) and pedestrian hit by a car (*n* = 1). Within the mechanisms of injury, there was a trend towards fewer traffic-related injuries throughout the study period and an increased ratio of falls (Fig. 3).

### Resource use

**Fig. 4:**
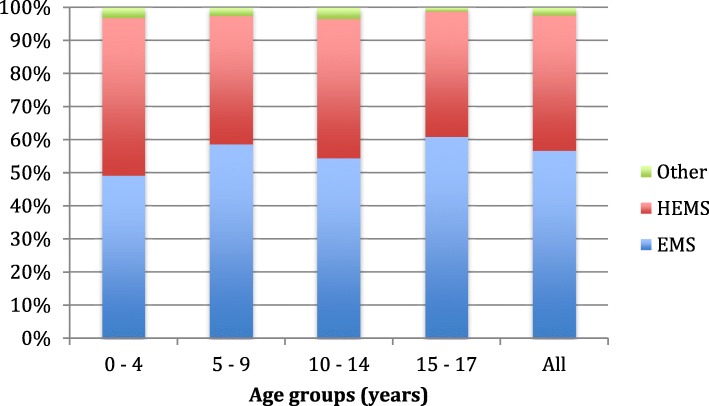
Mode of transport among different age groups. The figure illustrates the mode of transport among different age groups. There is a trend towards increased utilization of HEMS among the younger age groups. HEMS, Helicopter Emergency Medical System; EMS, Emergency Medical System

Fifty-seven per cent (*n* = 474) of the patients were transported to SOH by EMS and 41% (*n* = 341) by HEMS. Among the severely injured group, the main mode of transportation was by HEMS (60%, *n* = 68). The youngest age group (0–4 years) had a higher percentage of transportation by HEMS (48%) than the oldest age group (15–17 years, 38% by HEMS) (Fig. 4). The median length of hospital stay was 2 days in all patients, and 8 days among the severely injured patients. Twenty-three per cent (*n* = 199) of all patients stayed more than 1 day in the ICU, and 9% (*n* = 83) were treated one or more days on a ventilator. A CT scan was performed in 68% (*n* = 530) of cases, with 46% of these having pathological trauma-related findings. Fifty-four per cent (*n* = 325) of the patients in the minor injury group received some form of CT examination (head or chest/abdomen), of which 17% (*n* = 55) showed trauma-related findings. Pre-hospital intubation was performed in 54 patients (6%). Of the eight patients who died, five were intubated before reaching a hospital.

### Inter hospital transfers

Fifty-two patients (6%) were transferred from a local hospital to the trauma centre. Six patients were transferred by ambulance, 20 patients by HEMS, 3 by plane and the remaining 23 by an unknown mode of transportation. Forty-six per cent (*n* = 24) had severe injuries (ISS > 15), 37% (*n* = 19) had moderate injuries (ISS 9–15) and the remaining 17% (*n* = 9) had minor injuries (ISS < 9). None of the 52 patients transferred died within 30 days of the accident.

### Outcome

Eighty-three per cent (*n* = 723) of the included patients were reportedly discharged home after treatment at SOH. As many as 12% (*n* = 101) had an unknown discharge destination, 3% (*n* = 28) were transferred to a local hospital following discharge from SOH, less than 1% went to rehabilitation (*n* = 7) and less than 1% (*n* = 5) were transferred to a higher-level intensive care unit. Eight patients (1%) died within 30 days, while five patients had unknown survival data. One patient discharged to home and died within 30 days after injury.

## Discussion

This study shows that children constitute approximately 20% of all trauma patients admitted to our trauma centre. A small number of patients are characterized as severely injured, and only few patients die in the hospital following their injuries. The dominating type of injury is caused by blunt mechanism, with transport-related or falls being the most prevalent and with a tendency towards more transport-related injuries with increasing age. There is a trend towards increased use of HEMS with increasing injury severity and decreasing age. There are a low number of patients transferred from local hospitals, though almost half of these are severely injured.

Trauma is described by the World Health Organization as one of the great challenges of modern health care, representing one of the most common causes of death and disability from 1 to 44 years of age [1, 19]. There is a large variation between different parts of the world, with substantially higher rates of mortality in low-income countries [1, 20]. From 1990 to 2013, the overall consequences of road injuries among children decreased but ranked higher in 2013 (from 15 to 11) among the leading causes resulting in disability and death [20]. Our study shows that only a relatively small number of patients annually are considered potentially severely injured with an average of approximately 67 patients per year in a city population of 170,000 (Trondheim) and a regional population of 721,000 inhabitants. The annual number of severely injured children was low, meaning that very few physicians will obtain clinical experience with this patient group. Our findings are also consistent with national figures reporting a continuous decrease of fatally injured children in Norway since the 1980s [5, 21]. We found that many of the patients included were aged 15 years or older. These patients have similar physiology as adults, which limit the need for explicit paediatric expertise. Implementation of paediatric trauma teams, as seen in the USA, would therefore not seem like a cost-effective solution in Norway.

In our study, we used the ISS to define whether the patient had severe (ISS > 15), moderate (ISS 9–15) or minor (ISS 0–8) injuries. By using these definitions, only 15% of the patients included were defined as major trauma, resulting in an overtriage of 85%. Overtriage could be defined as the number of patients with minor trauma and receiving trauma team activation, whereas undertriage conversely would be severely injured patients not receiving trauma team activation [13]. The effects of overtriage would be over-utilization of costly resources, and undertriage could potentially lead to delayed treatment of patients with life-threatening injuries [13, 22]. In a study by Palmer et al., the use of a lower threshold of an ISS of 8 or higher was recommended as a better definition of a “severely injured” patient, specifically if both morbidity, mortality and the need of resources is to be assessed in paediatric trauma patients [23]. Considering this definition, a total of 33% of our patients could be defined as severely injured. We also reported NISS due to its increased predictive ability in penetrating trauma and isolated head injury and increased use in the literature [16]. In our study, a larger group of patients assessed with NISS could be defined as severely injured (NISS > 15), compared to ISS: 20% versus 15%. The clinical significance of this difference in our study is uncertain due to a low total number of severely injured patients, few deaths and short follow-up time.

We observed a total mortality rate of 1%, given that the patient is alive at hospital arrival and that a trauma team is activated. In the severely injured group, the mortality was 5%. This is lower than the mortality in our hospital among all severely injured patients, which is shown to be 12% [24]. Previous studies report that most paediatric deaths occur in the pre-hospital setting [9, 25]. Children have a different response to trauma than adults, and the injury pattern is often different [26]. We observed only small differences in physiological variables between the injury groups. This is of course limited by our large number of missing data on these variables and that normal ranges of vital parameters vary between the age groups, making it more difficult to define substantial differences. Children can compensate for injuries longer than adults compensate and sustain a normal blood pressure up until the moment they have a circulatory collapse [27]. When their vital signs ultimately are lost, attempts of rescue tend to be futile [28]. Head injuries are more common in children, due to anatomical differences. They are five times more likely to have respiratory problems caused by blunt trauma to the brain, then they are to have hypovolemic hypotension [26]. Physiological criteria for trauma team activation in children, especially circulatory variables, are therefore probably not as good an indicator of severe injury as in adults, and this age group needs a lower threshold for TTA.

The panorama of injuries in Scandinavia is different compared to many other countries, where penetrating injuries account for a much higher portion of injury mechanisms [8]. While we observed that only 2% of the patients had a penetrating trauma, studies from other parts of the world show 10 times as high percentage of penetrating trauma [28, 29]. Norway has a low incidence of gun and knife violence, and gun laws are strict. A low crime rate combined with a low availability of weapons could be the reason for penetrating trauma not being a big part of Norwegian paediatric trauma injuries [5]. Transport-related incidents are the most common cause of injuries, both globally and in Norway [1, 5]. This is also reflected within our findings, representing the patient selection of a single trauma centre. Transport-related traumas involving care were responsible for most of the minor injury patients. With new cars being safer, and having an exterior that implodes on impact to minimize forces applied to passengers, the dramatic sights on the scene of an accident may lead to a high overtriage. Globally, a marked reduction is observed in transport-related injuries [1]. The reasons for this may be due to several factors, but increased focus and use of injury prevention measures are likely to have contributed beneficially with a reduced number of injuries [5]. Although we did not observe any substantial reduction of patients throughout the study period, we observed a trend towards fewer transport-related injuries (Fig. 3), which could reflect the effects of targeted injury prevention measures. In other studies, the age group below 5 years, different major mechanisms of injury such as drowning and accidental suffocation have been reported [26]. In our study, we did not discriminate drowning or suffocation as a mechanism of injury but only registered them as “others”. It is unclear whether all near-drownings and suffocations trigger trauma team activation, and thereby would be eligible for inclusion in our study. In our patient cohort, two out of eight patients who died were reported with “others” as the mechanism of injury, and could therefore be drowning, suffocation or burn victims.

In our study, we also investigated the use of both pre- and in-hospital resources. We observed that there was a trend towards increased use of HEMS among younger age groups, also previously described [30]. In our setting, this could be explained by a lower threshold for using higher competence on the scene by bringing a specially trained anaesthesiologist to the scene, combined with a general low exposure of potentially severely injured children among EMS personnel and the need for rapid transport to a definitive care facility. Considering that our study cohort describes a 13-year time period, the use of ICU resources, performed key emergency surgical procedures and emergency procedures (e.g. intubation and thoracic drainage) remains low (Table 2). Therefore, the collective experience at this trauma centre and for each individual team member is sparse. Therefore, in order to compensate for this reduced in vivo experience of potentially severely injured children, systematic training seems warranted [31]. Similar challenges regarding low incidence and exposure to paediatric trauma are also seen in other countries [32, 33]. Introduction of targeted courses and training programmes provide greater confidence and better systematic approach to resuscitation, but the long-term effect on the outcome is still debated [32, 33]. A multiprofessional team-based approach to resuscitation of critically ill and injured children was initiated in Norway, but its effect on the outcome, system development and implementation has not yet been described [34]. Future research needs to focus on the effect of the implementation of systematic trauma care in children and its effect on the outcome.

We recognize some limitations in our study. First, due to inclusion criteria, those with severe injury and not treated by trauma teams (i.e. undertriage) were not included as no formal system within the hospital captured these patients. In the general trauma population, rates of undertriage from 10 to 19% have been described [13, 22, 35]. Second, the quality of the collected data is based on the precision of each registrar. As the components of our trauma system were developing during the study period of 13 years, several registrars were performing data sampling. Third, there was no formal regional trauma system or registry in place to ensure data capture on pre-hospital deaths; the study did not therefore include those patients who died before hospital arrival. Previous publications have described the rate of pre-hospital deaths ranging from 69 to 78% of all trauma deaths [36, 37].

## Conclusions

In a developed healthcare system, the number of potentially severely injured children is small and with very few deaths following trauma. Transport and falls represent the most common causes of injury throughout all age groups, though with a tendency towards more transport-related injuries with increasing age. In-hospital trauma care is characterized by a low threshold for a multidisciplinary reception, low use of intensive care and need for emergency surgical procedures, though with increased need in the older children.

## Additional file

Additional file 1: Table S1. Overview and quality of study variables (DOCX 15 kb)

## Data Availability

The datasets used during the current study are available from the corresponding author on reasonable request.

## Abbreviations

AIS: Abbreviated Injruy Scale
CT: Computer tomography
EMCC: Emergency Medical Coordination Centre
HEMS: Helicopter Emergency Medical Services
ICU: Intensive care unit
IQR: Interquartile range
ISS: Injury Severity Score
NISS: New Injury Severity Score
REC: Regional ethics committee
SOH: St. Olav’s University Hospital
STROBE: Strengthening the Reporting of Observational Studies in Epidemiology

## References

[1] Haagsma JA, Graetz N, Bolliger I, Naghavi M, Higashi H, Mullany EC (2016). The global burden of injury: incidence, mortality, disability-adjusted life years and time trends from the Global Burden of Disease study 2013. Inj Prev.

[2] Peden M, Oyegbite K, Ozanne-Smith J, Hyder AA, Branche C, Rahman A, Rivara F, Bartolomeos K, World Health Organization (2008). WHO: world report on child injury prevention. World report on child injury prevention.

[3] Morrison A, Stone DH (2000). Trends in injury mortality among young people in the European Union: a report from the EURORISC working group. J Adolesc Health.

[4] Brohi K, Cole E, Hoffman K (2011). Improving outcomes in the early phases after major trauma. Curr Opin Crit Care.

[5] The panorama of injuries in Norway - emphasis on injury in central registries [http://www.fhi.no/publ/2014/skadebildet-i-norge-hovedvekt-pa-pe2/]. Accessed 26 July 2019.

[6] Kristiansen T, Rehn M, Gravseth HM, Lossius HM, Kristensen P (2012). Paediatric trauma mortality in Norway: a population-based study of injury characteristics and urban-rural differences. Injury.

[7] Kristiansen T, Soreide K, Ringdal KG, Rehn M, Kruger AJ, Reite A, Meling T (2010). Trauma systems and early management of severe injuries in Scandinavia: review of the current state. Injury.

[8] Søreide K (2009). Epidemiology of major trauma. Br J Surg.

[9] Soreide K, Kruger AJ, Ellingsen CL, Tjosevik KE (2009). Pediatric trauma deaths are predominated by severe head injuries during spring and summer. Scand J Trauma Resusc Emerg Med.

[10] Norwegian Institute of Public Health. Mortality and causes of death in Norway through 60 years - 1951 – 2010. [http://www.fhi.no/publ/2012/dodelighet-og-dodsarsaker-i-norge-g/] Accessed 26 July 2019.

[11] von Elm E, Altman DG, Egger M, Pocock SJ, Gotzsche PC, Vandenbroucke JP (2007). The Strengthening the Reporting of Observational Studies in Epidemiology (STROBE) statement: guidelines for reporting observational studies. Bull World Health Organ.

[12] St. Olav’s University Hospital – about us [http://stolav.no/om-oss] Accessed 26 July 2019.

[13] Uleberg O, Vinjevoll OP, Eriksson U, Aadahl P, Skogvoll E (2007). Overtriage in trauma - what are the causes?. Acta Anaesthesiol Scand.

[14] Baker SP, O’Neill B, Haddon W, Long WB (1974). The injury severity score: a method for describing patients with multiple injuries and evaluating emergency care. J Trauma.

[15] Lavoie Andr??, Moore Lynne, LeSage Natalie, Liberman Moishe, Sampalis John S. (2004). The New Injury Severity Score: A More Accurate Predictor of In-Hospital Mortality than the Injury Severity Score. The Journal of Trauma: Injury, Infection, and Critical Care.

[16] Paffrath T, Lefering R, Flohe S, TraumaRegister DGU (2014). How to define severely injured patients? - an Injury Severity Score (ISS) based approach alone is not sufficient. Injury.

[17] Franzén L, Örtenwall P, Backteman T (2007). Children in Sweden admitted to intensive care after trauma. Injury.

[18] Boyd CR, Tolson MA, Copes WS (1987). Evaluating trauma care: the TRISS method. Trauma Score and the Injury Severity Score. J Trauma.

[19] World Health Organization. The injury chart book: a graphical overview of the global burden of injuries. Department of Injuries and Violence Prevention, Noncommunicable Diseases and Mental Health Cluster. Geneva: World Health Organization; 2002.

[20] Kyu HH, Pinho C, Wagner JA, Brown JC, Bertozzi-Villa A, Charlson FJ (2016). Global and national burden of diseases and injuries among children and adolescents between 1990 and 2013: findings from the Global Burden of Disease 2013 Study. JAMA Pediatr.

[21] Gjertsen F (1992). Accident mortality 1956-1998.

[22] Rehn M, Lossius HM, Tjosevik KE, Vetrhus M, Ostebo O, Eken T (2012). Efficacy of a two-tiered trauma team activation protocol in a Norwegian trauma centre. Br J Surg.

[23] Palmer C (2007). Major trauma and the injury severity score--where should we set the bar?. Annu Proc Assoc Adv Automot Med.

[24] Uleberg O, Kristiansen T, Pape K, Romundstad PR, Klepstad P (2017). Trauma care in a combined rural and urban region: an observational study. Acta Anaesthesiol Scand.

[25] Vane DW, Shackford SR (1995). Epidemiology of rural traumatic death in children: a population-based study. J Trauma.

[26] Walters Bryan S. (2014). Epidemiology of Trauma. Pediatric Surgery.

[27] Overly FL, Wills H, Valente JH (2014). ‘Not just little adults’ - a pediatric trauma primer. R I Med J.

[28] Allen CJ, Wagenaar AE, Horkan DB, Baldor DJ, Hannay WM, Tashiro J (2016). Predictors of mortality in pediatric trauma: experiences of a level 1 trauma center and an assessment of the International Classification Injury Severity Score (ICISS). Pediatr Surg Int.

[29] Cleves D, Gomez C, Davalos DM, Garcia X, Astudillo RE (2016). Pediatric trauma at a general hospital in Cali, Colombia. J Pediatr Surg.

[30] Cox S, Morrison C, Cameron P, Smith K (2014). Advancing age and trauma: triage destination compliance and mortality in Victoria, Australia. Injury.

[31] Wisborg T, Brattebo G, Brattebo J, Brinchmann-Hansen A (2006). Training multiprofessional trauma teams in Norwegian hospitals using simple and low cost local simulations. Educ Health (Abingdon).

[32] Ibáñez Pradas V, Pérez MR (2017). Quality of initial trauma care in paediatrics. An Pediatr (Barc).

[33] Williams D, Foglia R, Megison S, Garcia N, Foglia M, Vinson L (2011). Trauma activation: are we making the right call? A 3-year experience at a level I pediatric trauma center. J Pediatr Surg.

[34] Johannessen LB (2006). BEST – also when it comes to children. Norwegian. Tidsskr Nor Laegeforen.

[35] Rehn M, Eken T, Kruger AJ, Steen PA, Skaga NO, Lossius HM (2009). Precision of field triage in patients brought to a trauma centre after introducing trauma team activation guidelines. Scand J Trauma Resusc Emerg Med.

[36] Hansen KS, Morild I, Engesæter LB, Viste A (2004). Epidemiology of severely and fatally injured patients in western part of Norway. Scand J Surg.

[37] Kristiansen T, Lossius HM, Rehn M, Kristensen P, Gravseth HM, Roislien J (2014). Epidemiology of trauma: a population-based study of geographical risk factors for injury deaths in the working-age population of Norway. Injury.

