# Supplementary material for: Epidemiology of paediatric trauma in Norway: a single-trauma centre observational study
Source: Int J Emerg Med. 2019 Jul 31;12:18. doi: 10.1186/s12245-019-0236-9 (PMC6670199; doi:10.1186/s12245-019-0236-9)
Supplement: Supplementary file 1 — Table S1. Overview and quality of study variables (DOCX 15 kb) [file 12245_2019_236_MOESM1_ESM.docx]

Additional file 1: Table S1 - Overview and quality of study variables

| **Study variable** | **Valid values *n*** | **Missing values *n*** |
| --- | --- | --- |
| Date of injury | 873 | 0 |
| Time of injury | 834 | 39 |
| Age | 873 | 0 |
| Gender | 873 | 0 |
| Dominating type of injury | 870 | 3 |
| Mechanism of injury | 872 | 1 |
| Glasgow coma scale pre-hospital | 706 | 167 |
| Systolic blood pressure pre-hospital | 526 | 347 |
| Pulse pre-hospital | 530 | 343 |
| Respiratory rate pre-hospital | 500 | 373 |
| Glasgow coma scale upon arrival in ED | 839 | 34 |
| Systolic blood pressure upon arrival in ED | 797 | 76 |
| Pulse upon arrival in ED | 679 | 194 |
| Respiratory rate upon arrival in ED | 749 | 124 |
| Number of days on ventilator | 870 | 3 |
| Numbers of days in ICU | 869 | 4 |
| Number of days in main hospital | 865 | 8 |
| Discharged to | 772 | 101 |
| Survival status after 30 days after injury | 868 | 5 |
| Pre-hospital intubation | 830 | 43 |
| Thorax incision | 821 | 52 |
| Chest tube | 825 | 48 |
| Type of transportation | 836 | 37 |
| Inter-hospital transfer | 873 | 0 |
| Type of first key emergency intervention | 864 | 9 |
| CT taken at the hospital | 777 | 96 |
| CT caput taken at the hospital | 627 | 246 |
| CT Thorax/abdomen taken at the hospital | 617 | 256 |
| Pathologic CT | 647 | 226 |
| Injury Severity Score (ISS) | 873 | 0 |
| New Injury Severity Score (NISS) | 873 | 0 |
| Injury on horse | 786 | 87 |
| Injury on ski/sled | 787 | 86 |

**ED:** Emergency department; **ICU:** Intensive Care Unit; **CT:** Computer tomography
